# Decoding the Correlation Between Inflammatory Response Marker Interleukin-6 (IL-6) and C-reactive Protein (CRP) With Disease Activity in Rheumatoid Arthritis

**DOI:** 10.7759/cureus.62954

**Published:** 2024-06-23

**Authors:** Prajakta R Warjukar, Ankush V Mohabey, Pradeep B Jain, Gulshan R Bandre

**Affiliations:** 1 Biochemistry, Datta Meghe Medical College, Datta Meghe Institute of Higher Education and Research, Nagpur, IND; 2 Orthopedics, All India Institute of Medical Sciences, Nagpur, Nagpur, IND; 3 Microbiology, Jawaharlal Nehru Medical College, Datta Meghe Institute of Higher Education and Research, Wardha, IND

**Keywords:** inflammatory biomarkers, das28 score, rheumatoid arthritis, c-reactive protein, interleukin-6

## Abstract

Background: Excessive interleukin-6 (IL-6) production in rheumatoid arthritis (RA) leads to joint destruction, inflammation, and systemic symptoms. IL-6 inhibitors alleviate symptoms. C-reactive protein (CRP), an inflammation biomarker, correlates with RA activity. In this study, we assess IL-6 and CRP levels in RA patients to understand their association with disease activity.

Materials and methods: This cross-sectional study was conducted at a tertiary care hospital in central India for 15 months, from July 2022 to September 2023. The study involved 75 participants diagnosed with RA and receiving outpatient treatment. Exclusion criteria included anti-IL-6 drug treatment, bedridden individuals, proxy patients, and those without consent. Disease activity was assessed using the 28-joint disease activity score (DAS28), while IL-6 and CRP levels were measured following the standard procedures.

Results: The average CRP levels were found to be 51.67 ± 47.49 mg/L, while IL-6 levels averaged 65.16 ± 43.67 pg/ml. The results revealed a substantial positive correlation between IL-6 levels and DAS28 (r = 0.603, p value < 0.001), indicating a significant association. Additionally, a moderate correlation between CRP levels and DAS28 (r = 0.493, p value < 0.001) highlighted a significant relationship between these variables.

Conclusions: The analysis showed that higher IL-6 levels were associated with increased disease activity and suggested IL-6 as a valuable indicator for assessing RA severity. Also, CRP levels had a moderate correlation with disease activity. Overall, IL-6 is a better marker for disease activity when compared to CRP levels in patients with RA.

## Introduction

Rheumatoid arthritis (RA) is a chronic autoimmune disease characterized by ongoing joint inflammation resulting in pain, swelling, and joint damage [1,2]. Interlukin-6 (IL-6), a pro-inflammatory cytokine, influences the progression and severity of RA [3]. In individuals with RA, there is excessive production of IL-6 primarily by immune cells in the synovium [4,5]. The increased amounts of IL-6 are involved in the breakdown of joint tissues and continue the inflammatory reaction. Additionally, they contribute to the general symptoms of the condition, which encompass fatigue, reduced levels of red blood cells, and diminished appetite [5-7]. In addition, IL-6 promotes the development of new blood vessels (angiogenesis), increasing inflammation and pannus formation in the synovium [3]. As a result, focusing on IL-6 has become a recognized treatment strategy for controlling RA. Biological drugs like tocilizumab and sarilumab, known as IL-6 inhibitors, are utilized in this approach. By blocking the IL-6 receptor, these medications effectively decrease inflammation and relieve symptoms in individuals with moderate to severe RA. In addition, clinical studies have shown that these drugs effectively improve joint symptoms, prevent joint damage, and enhance physical functioning [8].

There are various markers used for diagnosis and management of RA like rheumatoid factor (RF), anti-cyclic citrullinated peptide (anti-CCP2), erythrocyte sedimentation rate (ESR), C-reactive protein (CRP), etc. CRP is one of the biomarkers used to assess and manage RA [9]. In response to inflammation occurring in different body areas, including the joints affected by RA, the liver produces the protein CRP. Therefore, increased levels of CRP in the blood indicate the presence and intensity of inflammation in RA [9-11]. CRP levels are closely linked to the activity of the disease and can be used to assess the effectiveness of treatment approaches. During active flare-ups of RA, CRP levels tend to be higher, while successful treatment or remission leads to a decrease in CRP levels [11,12]. The main objective of this study was to assess the concentrations of IL-6 and CRP in patients diagnosed with RA and examine how these levels relate to the activity of the disease.

## Materials and methods

This cross-sectional study was done at a tertiary care hospital in central India. The study was conducted over 15 months, from July 2022 to September 2023. The study comprised 75 participants who had been diagnosed with RA [13]* *and were receiving treatment at the outpatient medicine department. Exclusion criteria for the study included participants undergoing treatment with anti-IL-6 drugs for RA and other diseases, bedridden individuals, proxy patients, and those who did not provide consent. The sample size for the study was determined based on an estimated correlation of 0.35 between IL-6 levels and the 28-joint disease activity score (DAS28), with a 95% confidence interval and 80% power [14]. Accordingly, the minimum required sample size was calculated to be 62 participants. However, a total of 75 cases were included in this study. The magnitude of the coefficient correlation was classified as per Schober et al. [15].

DAS28

In this study, we calculated DAS28 by considering the number of swollen or tender joints. Based on their DAS28, the patients were classified into four groups: remission (DAS28 < 2.6), mild (2.6 ≤ DAS28 < 3.2), moderate (3.2 ≤ DAS28 < 5.1), and severe (DAS28 ≥ 5.1). For individuals with a DAS28 higher than 5.1, a follow-up assessment of DAS28 was conducted and blood samples were collected to investigate any potential relationship between serum cytokine levels and disease activity [10,16]. In addition, blood samples were taken during the clinical evaluation for disease activity to measure the levels of CRP and IL-6.

Sample collection and testing procedure

To analyze the serum samples, they were obtained intravenously and subjected to centrifugation. Subsequently, the samples were stored at 2-8 °C until further analysis. The serum level of IL-6 was determined using a commercial chemiluminescent immunoassay kit. This IL-6 assay utilized a one-step immuno-enzymatic method known as the "sandwich" method. First, the sample was mixed with paramagnetic particles coated with mouse monoclonal anti-human IL-6, blocking reagent, and alkaline phosphatase conjugate. After incubation, materials attached to the solid phase were retained using a magnetic field, while unbound materials were washed away. Then, the vessel was treated with a chemiluminescent substrate Lumi-Phos*530 (Lumigen, Inc., Southfield, USA), and the resulting light produced by the reaction was measured using a luminometer. The amount of light generated directly correlated with the concentration of IL-6 in the sample. Finally, the quantity of IL-6 in the sample was determined based on a stored calibration curve with multiple data points. As per a non-parametric reference interval analysis, the IL-6 upper 95% interval of the reference range was <6.4 pg/mL [17]. A commercial CRP kit was used to measure CRP, employing the immunoturbidimetric technique. This study's reference range for CRP was stated as less than 5 mg/L [18].

Ethical considerations

Before initiating the study, all participants were provided with written consent forms, which ensured that they received comprehensive information about the study and willingly agreed to take part. Throughout the study, strict confidentiality regarding their personal information was maintained. Prior approval was obtained for the study from the Institutional Ethics Committee before initiation vide letter number SMHRC/IEC/2022/12-16.

Statistical analysis

The data was gathered, organized, and analyzed utilizing EPI Info version 7.2 (Centers for Disease Control and Prevention, Atlanta, USA). Qualitative variables were presented as percentages while quantitative variables were categorized and expressed as percentages, means, and standard deviations. The disparity between the two proportions was examined using the chi-square or Fisher's exact tests. All analyses were performed with a two-tailed approach, and a significance level of 0.05 was established.

## Results

We included 75 cases in the present study of which 62 were female and 13 were male subjects. Table 1 shows that the patients had an average age of 39.45 ± 8.23 years and the duration of the disease was 3.1 ± 0.78 years. The mean CRP levels were 51.67 ± 47.49 mg/L and the IL-6 levels averaged 65.16 ± 43.67 pg/mL.

**Table 1:** Demographic particulars of the present sample SD: Standard deviation; IL: Interleukin; CRP: C-reactive protein

Demographic particulars	Mean ± SD
Age (years)	39.45 ± 8.23
Duration of disease (years)	3.1 ± 0.78
CRP levels (mg/L)	51.67 ± 47.49
IL-6 levels (pg/mL)	65.16 ± 43.67

Table 2 shows that out of the 75 cases analyzed 15 (20%) had a DAS28 below 2.6, seven (9.33%) had a score between 2.6 and 3.2, 40 (53.33%) had a score between 3.2 and 5.1, and 13 (17.33%) had a score exceeding 5.1.

**Table 2:** Distribution of 75 patients based on DAS28 DAS28: 28-joint disease activity score

DAS28	Number of patients (%)
<2.6	15 (20%)
2.6-3.2	7 (9.33%)
3.2-5.1	40 (53.33%)
>5.1	13 (17.33%)

**Figure 1:**
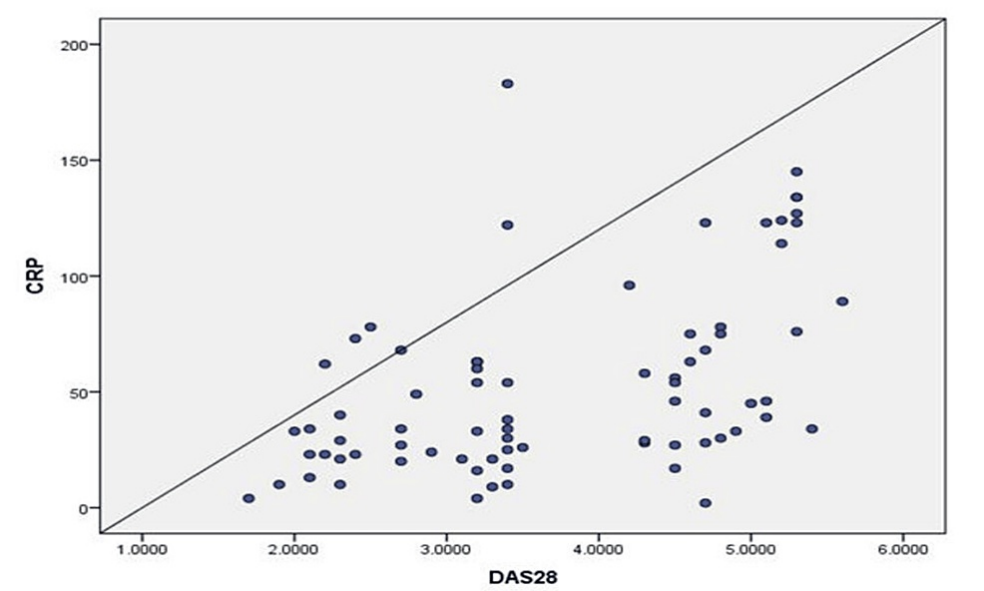
Scatter diagram showing the correlation of CRP with DAS28 score DAS28: Disease activity score; CRP: C-reactive protein

**Figure 2:**
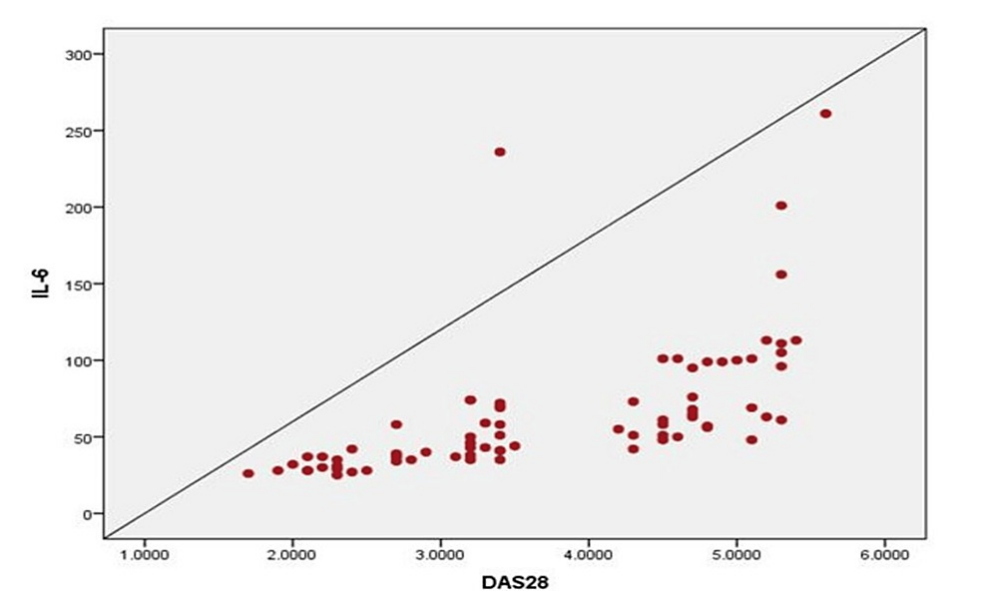
Scatter diagram showing the correlation of IL-6 with DAS28 DAS28: Disease activity score; IL-6: Interleukin-6

Table 3 shows a moderate correlation between CRP levels and disease activity (r = 0.493, p value < 0.001) (Figure 1), indicating a significant association. Furthermore, there was a strong positive correlation between IL-6 levels and DAS28 (r = 0.603, p value < 0.001), suggesting a notable relationship between these variables (Figure 2).

**Table 3:** Correlation of IL-6 and CRP levels with DAS28 DAS28: Disease activity score; IL: Interleukin; CRP: C-reactive protein P value <0.05: Significant value

DAS28	r value	P value
IL-6	0.603	<0.001
CRP	0.493	<0.001

## Discussion

RA is a chronic autoimmune disorder characterized by joint inflammation, leading to pain, stiffness, and swelling symptoms. While the exact cause of RA remains unknown, it is believed to arise from genetic and environmental factors. The disease can progress without proper treatment and result in joint deformity and impaired functionality [1,19]. Hence, it is crucial to initiate early and proactive measures to attain remission, decrease disease activity, and prevent potential complications in RA. Monitoring disease activity and adjusting treatment approaches are pivotal in effective management. Researchers have investigated the correlation between disease activity in RA and specific markers such as IL-6 and CRP, providing valuable insights for clinical assessment and treatment decisions [6,20].

Research studies have demonstrated a positive association between IL-6 levels and disease activity, as assessed by DAS28. For example, Wei et al. [21] discovered a significant positive correlation between IL-6 levels and disease activity in individuals diagnosed with RA. Similarly, Nishimoto et al. [22] observed elevated IL-6 levels in patients with active disease compared to those in remission, suggesting IL-6 as a potential biomarker for monitoring disease activity. A meta-analysis conducted by Boyapati et al. [23] supported these findings, confirming a link between higher IL-6 levels and increased disease severity. IL-6 is recognized for its crucial role in the inflammatory response and is implicated in developing various inflammatory conditions including RA. The concentration of soluble IL-6 receptors may influence the inflammatory or anti-inflammatory functions of IL-6.

CRP levels are also positively correlated with disease activity in RA. In a systematic review, Navarro et al. [24] presented consistent evidence supporting the positive association between CRP levels and disease activity measures such as the DAS28 and clinical disease activity index (CDAI). CRP has been recognized as a valuable biomarker for monitoring disease activity in RA. Furthermore, a prospective cohort study by Yildirim et al. [25], demonstrated that CRP levels were linked to changes in disease activity over time, further validating its usefulness as a dynamic marker for tracking disease progression.

Since our study was designed as a cross-sectional study, it provides information on the relationship between IL-6, CRP, and disease activity at a single point in time, and we could not investigate how IL-6 and CRP levels vary with disease activity at different stages of treatment. As the study population was taken from a single center, it may not represent the broader RA population, which may restrict the generalizability of the findings of the study. Multicentric and longitudinal studies may offer more information to explore the correlation between symptom profiles and these markers. Nevertheless, this study represents a pioneering effort conducted in our geographic region to explore the relationship between disease activity and IL-6 and CRP levels.

## Conclusions

The analysis conducted in this study reveals a significant association between higher IL-6 levels and increased disease activity in patients with RA. This finding indicates that IL-6 may serve as a valuable indicator for assessing the severity of RA. In addition, IL-6, known for its involvement in inflammatory response, has been implicated in the pathogenesis of RA and is believed to contribute to the destruction of joint tissues and the perpetuation of the inflammatory process. On the other hand, CRP levels demonstrated a moderate correlation with disease activity. CRP, produced by the liver in response to inflammation, has long been recognized as a marker of systemic inflammation. While CRP levels can provide insight into the presence and severity of inflammation in RA, the correlation with DAS28 is less strong than that observed with IL-6. Based on the results of this study, IL-6 is a more reliable marker for assessing disease activity in patients with RA compared to CRP levels. Therefore, IL-6 measurement could be a valuable tool in clinical practice for monitoring disease progression, evaluating treatment response, and making informed decisions regarding disease management. Further research is warranted to explore the mechanisms underlying the relationship between IL-6, CRP, and disease activity in RA. Additionally, longitudinal studies may shed light on the dynamic changes in IL-6 and CRP levels over time and their implications for disease monitoring and treatment optimization in RA patients.

## References

[1] Heidari B (2011). Rheumatoid arthritis: early diagnosis and treatment outcomes. Caspian J Intern Med.

[2] Lin YJ, Anzaghe M, Schülke S (2020). Update on the pathomechanism, diagnosis, and treatment options for rheumatoid arthritis. Cells.

[3] Yoshida Y, Tanaka T (2014). Interleukin 6 and rheumatoid arthritis. Biomed Res Int.

[4] Tanaka T, Narazaki M, Kishimoto T (2014). IL-6 in inflammation, immunity, and disease. Cold Spring Harb Perspect Biol.

[5] Hashizume M, Mihara M (2011). The roles of interleukin-6 in the pathogenesis of rheumatoid arthritis. Arthritis.

[6] Pandolfi F, Franza L, Carusi V, Altamura S, Andriollo G, Nucera E (2020). Interleukin-6 in rheumatoid arthritis. Int J Mol Sci.

[7] Srirangan S, Choy EH (2010). The role of interleukin 6 in the pathophysiology of rheumatoid arthritis. Ther Adv Musculoskelet Dis.

[8] Yip RM, Yim CW (2021). Role of interleukin 6 inhibitors in the management of rheumatoid arthritis. J Clin Rheumatol.

[9] Shapiro SC (2021). Biomarkers in rheumatoid arthritis. Cureus.

[10] Orr CK, Najm A, Young F, McGarry T, Biniecka M, Fearon U, Veale DJ (2018). The utility and limitations of CRP, ESR and DAS28-CRP in appraising disease activity in rheumatoid arthritis. Front Med (Lausanne).

[11] Shadick NA, Cook NR, Karlson EW (2006). C-reactive protein in the prediction of rheumatoid arthritis in women. Arch Intern Med.

[12] Pope JE, Choy EH (2021). C-reactive protein and implications in rheumatoid arthritis and associated comorbidities. Semin Arthritis Rheum.

[13] Aletaha D, Neogi T, Silman AJ (2010). 2010 rheumatoid arthritis classification criteria: an American College of Rheumatology/European League Against Rheumatism collaborative initiative. Arthritis Rheum.

[14] Johnson TM, Register KA, Schmidt CM, O'Dell JR, Mikuls TR, Michaud K, England BR (2019). Correlation of the multi-biomarker disease activity score with rheumatoid arthritis disease activity measures: a systematic review and meta-analysis. Arthritis Care Res (Hoboken).

[15] Schober P, Boer C, Schwarte LA (2018). Correlation coefficients: appropriate use and interpretation. Anesth Analg.

[16] van Riel PL, Renskers L (2016). The disease activity score (DAS) and the disease activity score using 28 joint counts (DAS28) in the management of rheumatoid arthritis. Clin Exp Rheumatol.

[17] Dati F, Schumann G, Thomas L Access IL-6 - IFU. Eur J Clin Chem Clin Biochem.

[18] Dati F, Schumann G, Thomas L (1996). Consensus of a group of professional societies and diagnostic companies on guidelines for interim reference ranges for 14 proteins in serum based on the standardization against the IFCC/BCR/CAP Reference Material (CRM 470). Eur J Clin Chem Clin Biochem.

[19] Guo Q, Wang Y, Xu D, Nossent J, Pavlos NJ, Xu J (2018). Rheumatoid arthritis: pathological mechanisms and modern pharmacologic therapies. Bone Res.

[20] Rodriguez-García SC, Montes N, Ivorra-Cortes J (2021). Disease activity indices in rheumatoid arthritis: comparative performance to detect changes in function, IL-6 levels, and radiographic progression. Front Med (Lausanne).

[21] Wei ST, Sun YH, Zong SH, Xiang YB (2015). Serum levels of IL-6 and TNF-α may correlate with activity and severity of rheumatoid arthritis. Med Sci Monit.

[22] Nishimoto N, Miyasaka N, Yamamoto K, Kawai S, Takeuchi T, Azuma J, Kishimoto T (2009). Study of active controlled tocilizumab monotherapy for rheumatoid arthritis patients with an inadequate response to methotrexate (SATORI): significant reduction in disease activity and serum vascular endothelial growth factor by IL-6 receptor inhibition therapy. Mod Rheumatol.

[23] Boyapati A, Schwartzman S, Msihid J (2020). Association of high serum interleukin-6 levels with severe progression of rheumatoid arthritis and increased treatment response differentiating sarilumab from adalimumab or methotrexate in a post hoc analysis. Arthritis Rheumatol.

[24] Navarro-Compán V, Gherghe AM, Smolen JS, Aletaha D, Landewé R, van der Heijde D (2015). Relationship between disease activity indices and their individual components and radiographic progression in RA: a systematic literature review. Rheumatology (Oxford).

[25] Yildirim K, Karatay S, Melikoglu MA, Gureser G, Ugur M, Senel K (2004). Associations between acute phase reactant levels and disease activity score (DAS28) in patients with rheumatoid arthritis. Ann Clin Lab Sci.

